# Nivolumab-Induced Autoimmune Encephalitis in Two Patients with Lung Adenocarcinoma

**DOI:** 10.1155/2018/2548528

**Published:** 2018-07-04

**Authors:** Suma Shah, Anastasie Dunn-Pirio, Matthew Luedke, Joel Morgenlander, Mark Skeen, Christopher Eckstein

**Affiliations:** ^1^Duke University Department of Neurology, USA; ^2^Duke University Departments of Neurology and Orthopedic Surgery, USA

## Abstract

Immune checkpoint inhibitors have improved patient survival outcomes in a variety of advanced malignancies. However, they can cause a number of immune-related adverse effects (irAEs) through lymphocyte dysregulation. Central nervous system (CNS) irAEs are rare, but as the number of indications for checkpoint inhibitors increases, there has been emergence of CNS immune-mediated disease among cancer patients. Given the relatively recent recognition of checkpoint inhibitor CNS irAEs, there is no standard treatment, and prognosis is variable. Therefore, there is a great need for further study of checkpoint inhibitor-induced CNS irAEs. Here, we present two unique cases of nivolumab-induced autoimmune encephalitis in patients with non-small cell lung cancer and review the available literature.

## 1. Introduction

Immune checkpoints are built-in regulatory mechanisms of the adaptive immune system that function to maintain self-tolerance and attenuate physiologic immune responses [1]. Tumors can evade immune surveillance by manipulating immune checkpoints to establish more favorable environments for their growth [2]. Landmark clinical trials of immune checkpoint inhibitors targeting cytotoxic T-lymphocyte associated protein 4 (CTLA4) and the programmed cell death protein 1 (PD-1)/programmed death-ligand 1 (PD-L1) pathway have demonstrated improved survival rates in a variety of advanced malignancies [3].

Although checkpoint inhibitors have proven efficacy and are welcomed alternatives to traditional cytotoxic chemotherapy, they can cause immune-related adverse events (irAEs) due to their interference with lymphocyte regulation. Commonly described irAEs include rash, pruritus, colitis, hepatitis, and various endocrinopathies, such as thyroiditis and hypophysitis [4]. Neurologic irAEs are far less frequent and most often involve the peripheral nervous system [5, 6]. Here, we present two unique cases of central nervous system (CNS) irAEs following treatment with PD-1 inhibitor, nivolumab.

## 2. Case 1

A 66-year-old Caucasian woman with stage IIIb lung adenocarcinoma developed right hemiballismus and dysarthria following four months of nivolumab administration. The hemiballismus then evolved to bilateral ballismus in all extremities over a two-week period. Neurologic examination revealed hypophonic and dysarthric speech, orobuccolingual dyskinesias, and severe bilateral arm and leg ballismus.

**Figure 1:**
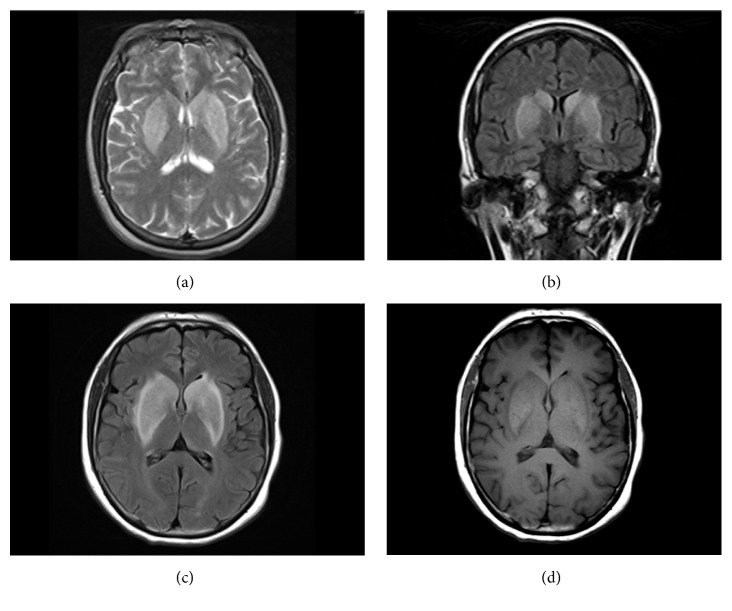
Initial and follow-up MRI brain for Case*    *1. (a) Initial MRI: axial T2-weighted image with hyperintensities in the bilateral basal ganglia. (b) Initial MRI: coronal FLAIR-weighted image with hyperintensities in the bilateral basal ganglia. (c) Follow-up MRI: axial FLAIR-weighted image with hyperintensities in the bilateral basal ganglia. (d) Follow-up MRI: axial T1-weighted image with hyperintensities in the bilateral basal ganglia.

Initial brain magnetic resonance imaging (MRI) with and without gadolinium showed symmetric T2 hyperintense and T1 hypointense basal ganglia abnormalities [Figures 1(a) and 1(b)]. Cerebrospinal fluid (CSF) analysis demonstrated a normal cell count and glucose level, a mildly elevated protein concentration of 56mg/dL (15-50mg/dL), and negative cytology. There were 16 oligoclonal bands present in the CSF compared to 2 in the serum. A CSF paraneoplastic antibody assay revealed a novel, unclassified antibody. A repeat brain MRI three weeks later redemonstrated symmetric T2 hyperintense basal ganglia but with a transition to T1 hyperintensities in the same location [Figures 1(c) and 1(d)].

Despite the consensus of an immune-mediated etiology, the patient was refractory to 5 days of intravenous (IV) methylprednisolone (1000mg/day) and 5 plasma exchanges. Haloperidol and olanzapine also did not offer symptomatic relief. She continued to decline despite subsequent trials of IV immunoglobulin (IVIg) (total dose of 2.5g/kg), prednisone, rituximab (1000mg once), and tetrabenazine (20mg, 3x/day). Due to continued clinical decline, she was eventually transitioned to comfort-only care and inpatient hospice.

## 3. Case 2

A 44-year-old Caucasian woman with type 1 diabetes mellitus (DM1) diagnosed at age 30 and stage IV lung adenocarcinoma treated with 5 cycles of nivolumab (3 mg/kg, every 2 weeks) developed several days of progressive altered mental status, nausea, and vomiting. She then presented to the emergency department following a first time seizure. Upon initial evaluation, she exhibited abnormal tongue movements, inappropriate laughter, and rhythmic movements of her right arm that improved with lorazepam.

**Figure 2:**
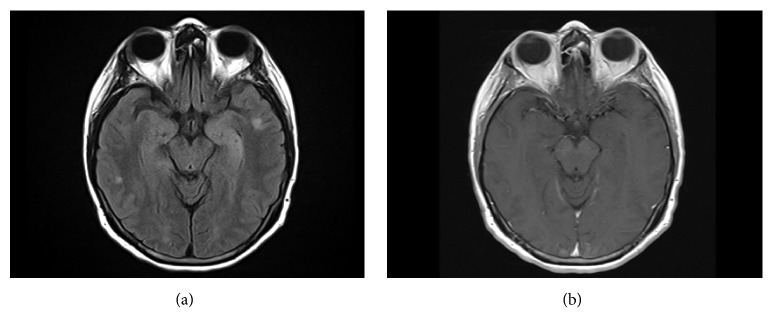
MRI brain imaging for Case*    *2. (a) MRI brain FLAIR imaging. This image demonstrates mildly expansile T2 signal hyperintensity of the left greater than right mesial temporal lobes. Additional small regions of cortical and subcortical T2 signal hyperintensity are noted in the temporal lobes of both hemispheres. (b) MRI brain, T1 sequence with contrast. There is no enhancement noted in the affected areas after administration of gadolinium contrast.

An electroencephalogram revealed left temporal slowing and frequent interictal discharges. Brain MRI with and without gadolinium demonstrated T2 signal hyperintensities of the bilateral mesial temporal lobes compatible with limbic encephalitis. Additionally, there were 2 enhancing foci within the left occipital and right temporal lobes, concerning for metastatic disease [Figure 2]. CSF analysis detected 19 nucleated cells (97% lymphocytes) and normal protein and glucose levels. There were 7 oligoclonal bands in the CSF and 3 in the serum. CSF cytology was negative. A CSF autoimmune encephalitis panel (Mayo Medical Laboratories) demonstrated the presence of glutamic acid decarboxylase 65-isoform (GAD65) antibodies: 2.70nmol/L (<= 0.02nmol/L). Serum GAD65 antibodies were also detected: 275nmol/L (<= 0.02nmol/L).

The patient was diagnosed with GAD65 antibody positive autoimmune encephalitis. She received IV methylprednisolone (1000mg/day) for 5 days followed by 5 plasma exchanges. However, she continued to experience refractory seizures despite treatment with multiple antiepileptic drugs and developed worsening ataxia, vertigo, and gait impairment. Therefore, she was given IV rituximab (1000mg) during the hospitalization. Upon discharge, seizures were under control and mental status improved. The patient currently receives maintenance rituximab (1000mg) every 6 months and remains seizure-free but with severe residual vertigo and moderate gait ataxia. Her most recent brain MRI demonstrated interval resolution of enhancing foci and abnormal T2 signal in the temporal lobes [Figure 2]. Following discontinuation of nivolumab, she was transitioned to brigatinib (a multikinase inhibitor with activity against anaplastic lymphoma kinase (ALK) as well as EGFR deletions and point mutations) for lung cancer treatment and remains oncologically stable.

## 4. Discussion

Systemic irAEs secondary to immune checkpoint blockade are a well-recognized phenomenon. Most systemic irAEs are successfully managed by discontinuing the offending agent alone or in combination with temporary immunosuppressive therapy such as corticosteroids and/or tumor necrosis factor-alpha (TNF-*α*) inhibitors [4]. In contrast, neurological irAEs occur less frequently, estimated in < 1% of individuals receiving immune checkpoint inhibitors. They can have more aggressive clinical courses and often involve the peripheral nervous system, especially the neuromuscular junction [6, 7]. In fact, clinical trial data indicate that autoimmune encephalitis occurs in as few as 0.1 to <1% of patients receiving checkpoint inhibition [8].

To the best of our knowledge, our cases represent the first descriptions of nivolumab-induced autoimmune encephalitis manifesting as choreiform movements as well as a GAD65 antibody positive autoimmune encephalitis. Each case entailed an aggressive neurological disease course, with the patient in the former case ultimately succumbing to the irAE. This is particularly troubling because development of nivolumab-induced irAEs in patients with non-small cell lung cancer (NSCLC) may predict a favorable oncologic treatment response [9].

It is to be determined whether these cases represent checkpoint inhibitor-induced de novo autoimmunity or the unmasking of a preexisting subclinical disorder. In oncology, the study of PD-1/PD-L1 signaling has largely focused on PD-L1 expression within the tumor microenvironment resulting in PD-1+T-lymphocyte inhibition and subsequent tumor escape. However, the role of PD-1/PD-L1 interactions extends beyond cell-mediated immunity and into humoral immunity. Specifically, PD-1+ follicular helper T cells (T_FH_ cells) are critical for germinal center function and antibody production. It was recently demonstrated that PD-L1+ regulatory B cells negatively regulate T_FH_ cells and thus attenuate humoral responses [10]. Therefore, it is possible that disruption of the PD-1/PD-L1 interaction at the level of the germinal center may have led to the de novo formation of aberrantly directed antibodies to self-antigens found in the CNS.

We suspect that the patient in Case 2, who was a known type 1 diabetic before developing nivolumab-associated GAD65 autoimmune encephalitis, was already at risk for developing CNS autoimmunity. As GAD65 is located on both pancreatic islet cells and CNS gamma-amino-butyric acid (GABA-ergic) neurons, there is an association between GAD65 antibody positive CNS autoimmune disease and DM1. Antibodies against GAD65 are detectable in roughly 70-80% of individuals with DM1 but typically at lower titers than in individuals who have coexisting neurologic autoimmunity [11, 12]. If the patient had preexisting GAD65 antibodies, it is plausible that nivolumab exposure simply exacerbated her condition. If more GAD65 antibody positive CNS autoimmunity are reported in patients with type 1 diabetes following immune checkpoint blockade, then screening for preexisting GAD65 antibodies prior to cancer immunotherapy may be clinically useful.

As the development of irAEs from nivolumab in patients with NSCLC has been correlated with improved cancer-related outcomes it is crucial to aggressively manage irAEs as well as supporting patients through the acute immune-mediated illness so they can continue with appropriate cancer treatment. Unlike the more common systemic irAEs, CNS irAEs are rare, and, therefore, optimal treatment is not as well established. In addition to establishing more efficacious treatments for CNS irAEs, developing biomarkers to predict risk of developing CNS irAEs is also warranted.

## References

[1] Pardoll D. M. (2012). The blockade of immune checkpoints in cancer immunotherapy. *Nature Reviews Cancer*.

[2] Diesendruck Y., Benhar I. (2017). Novel immune check point inhibiting antibodies in cancer therapy—Opportunities and challenges. *Drug Resistance Updates*.

[3] Wilson R. A. M., Evans T. R. J., Fraser A. R., Nibbs R. J. B. (2018). Immune checkpoint inhibitors: New strategies to checkmate cancer. *Clinical & Experimental Immunology*.

[4] Postow M. A., Sidlow R., Hellmann M. D. (2018). Immune-related adverse events associated with immune checkpoint blockade. *The New England Journal of Medicine*.

[5] Naidoo J., Page D. B., Li B. T. (2015). Toxicities of the anti-PD-1 and anti-PD-L1 immune checkpoint antibodies. *Annals of Oncology*.

[6] Makarious D., Horwood K., Coward J. I. G. (2017). Myasthenia gravis: An emerging toxicity of immune checkpoint inhibitors. *European Journal of Cancer*.

[7] Larkin J., Chmielowski B., Lao C. D. (2017). Neurologic serious adverse events associated with nivolumab plus ipilimumab or nivolumab alone in advanced melanoma, including a case series of encephalitis. *The Oncologist*.

[8] Schneider S., Potthast S., Komminoth P., Schwegler G., Böhm S. (2017). PD-1 checkpoint inhibitor associated autoimmune encephalitis. *Case Reports in Oncology*.

[9] Sato K., Akamatsu H., Murakami E. (2018). Correlation between immune-related adverse events and efficacy in non-small cell lung cancer treated with nivolumab. *Lung Cancer*.

[10] Khan A. R., Hams E., Floudas A., Sparwasser T., Weaver C. T., Fallon P. G. (2015). PD-L1hi B cells are critical regulators of humoral immunity. *Nature Communications*.

[11] McKeon A., Tracy J. A. (2017). GAD65 neurological autoimmunity. *Muscle & Nerve*.

[12] Notkins A. L., Lernmark Å. (2001). Autoimmune type 1 diabetes: Resolved and unresolved issues. *The Journal of Clinical Investigation*.

